# Evidence of the Presence of a Functional Dot/Icm Type IV-B Secretion System in the Fish Bacterial Pathogen *Piscirickettsia salmonis*

**DOI:** 10.1371/journal.pone.0054934

**Published:** 2013-01-28

**Authors:** Fernando A. Gómez, Jaime A. Tobar, Vitalia Henríquez, Mariel Sola, Claudia Altamirano, Sergio H. Marshall

**Affiliations:** 1 Laboratorio de Genética e Inmunología Molecular, Instituto de Biología, Pontificia Universidad Católica de Valparaíso, Valparaíso, Chile; 2 Núcleo de Biotecnología Curauma, Pontificia Universidad Católica de Valparaíso, Valparaíso, Chile; 3 Investigación y Desarrollo Biológicos, Laboratorio Centrovet, Santiago, Chile; 4 Fraunhofer Chile Research Foundation, Center For Systems Biotechnology, Santiago, Chile; 5 Escuela de Ingeniería Bioquímica, Pontificia Universidad Católica de Valparaíso, Valparaíso, Chile; University of Padova, Italy

## Abstract

*Piscirickettsia salmonis* is a fish bacterial pathogen that has severely challenged the sustainability of the Chilean salmon industry since its appearance in 1989. As this Gram-negative bacterium has been poorly characterized, relevant aspects of its life cycle, virulence and pathogenesis must be identified in order to properly design prophylactic procedures. This report provides evidence of the functional presence in *P. salmonis* of four genes homologous to those described for Dot/Icm Type IV Secretion Systems. The Dot/Icm System, the major virulence mechanism of phylogenetically related pathogens *Legionella pneumophila* and *Coxiella burnetii*, is responsible for their intracellular survival and multiplication, conditions that may also apply to *P. salmonis*. Our results demonstrate that the four *P. salmonis dot/icm* homologues (*dotB, dotA, icmK* and *icmE*) are expressed both during *in vitro* tissue culture cells infection and growing in cell-free media, suggestive of their putative constitutive expression. Additionally, as it happens in other referential bacterial systems, temporal acidification of cell-free media results in over expression of all four *P. salmonis* genes, a well-known strategy by which SSTIV-containing bacteria inhibit phagosome-lysosome fusion to survive. These findings are very important to understand the virulence mechanisms of *P. salmonis* in order to design new prophylactic alternatives to control the disease.

## Introduction

*Piscirickettsia salmonis* is an aggressive facultative intracellular bacterium that severely threatens the sustainability of the salmon industry in Chile. The bacterium is known to be the etiological agent of Salmonid Rickettsial Septicaemia (SRS) or Piscirickettsiosis [1], a disease that produces systemic infection characterized by colonization of several organs including kidney, liver, spleen, intestine, brain, ovary and gills [1], [2]. Since it was first reported in Chile in 1989 [3], SRS has been reported in other latitudes but with significantly less impact [4], [5]. *P. salmonis* belongs to the group of Gamma proteobacteria [6], [7] and to date has been described as non-motile, non-encapsulated, pleomorphic but generally cocoid, with a diameter from 0.1 to 1.5 µm [8], [9], [10]. The bacterium was first described as an obligate intracellular pathogen and today is able to grow in cell-free media [11], [12], [13], [14]. *P. salmonis* productively infect salmonids and other economically important fish species [15], [16], [17]. This also confirms that the expected spread of the agent to other commercially relevant fish species has already started and is a clear indication of its potential threat [18]. Despite the high impact *P. salmonis* has had on the aquaculture industry, key aspects of its biology, pathogenesis and virulence are completely unknown, a situation that has significantly hampered control [19].

It was recently reported that *P. salmonis* infects macrophages by multiplying inside replicative vacuoles [10], [20], [21] and also induces apoptosis via caspase-3 activation in these cells [22]. Macrophage infection is a common strategy adopted by several intracellular pathogens to colonize and systemically spread into their hosts by using different molecular effectors to interfere with normal cell signaling and thus disable normal responses triggered to control and eliminate foreign invaders. One of the most important of these responses is lysosome-mediated degradation, which if impeded, allows the invader to succeed in its infection [23], [24]. After infection, phagosome acidification is an initial cell response that is essential to avoiding intracellular multiplication of many pathogenic organisms including *Mycobacterium, Chlamydia spp., L. pneumophila, C. burnetii,* and *B.suis* [25], [26]. The bacteria in turn react by isolating themselves in vacuoles inside the infected cell to avoid fusion of the acidified phagosome with a degrading lysosome [27]. In order to do that the bacteria inside the phagosome express different effectors molecules coded by different secretion system genes that promotes their replication [28].

In pathogenic bacteria, most of the seven secretion systems (SSs) described are conserved machineries involved in the secretion of virulence effectors as well as other molecules that tend to undermine key host-cell functions for allowing the pathogen to establish permissive niches to survive [29]. One of the most significant undermining targets of SSs is to impede phagosome-lysosome fusion. This distinctive feature is particularly enhanced by the different variants of Type IV Secretion Systems (TIVSSs). TIVSSs are one of many mechanisms widely used by various intracellular and non-intracellular pathogens [30], [31], [32]. In addition, TIVSSs are highly versatile as they are able to secrete not only proteins, such as the *Helicobacter pylori* Cag-type IVsecretion system [33], [34], but also DNA, as reported for *Agrobacterium tumefaciens* VirB/D4 system. *Legionella pneumophila* and *Coxiella burnetii*, two important human pathogens, use a particular TIVSS named Dot/Icm (Deficient in Organelle Trafficking/Intracellular Multiplication) to establish productive infections [35], [36]. The Dot/Icm system comprises around 20 different proteins including DotB and DotA as the most significant ones [35].The mimicking of *Legionella pneumophila*'s system could represent one of the important components of the bacterial virulence for *P. salmonis* where the macrophage infections are preferred target for the multiplication of replicative vacuoles [37]. Furthermore, the *L. pneumophila* Dot/Icm system is also involved in phagocytosis, cytotoxicity, apoptosis and also in inhibition of phagosome-lysosome fusion which leads to the formation of a novel ribosome-lined phagosome [38], all of these may likewise be expected to occur in *P. salmonis.* We focus our attention on the experimental determination of whether *P. salmonis* possesses the Dot/Icm secretion system or an equivalent one for achieving productive infection, since *P. salmonis* is phylogenetically related to *L. pneumophila* and *C. burnetti*, assuming as well that they may share similar pathways to infect susceptible host cells. We have found four *dot/icm* homologues in the genome of *P. salmonis* by using PCR-based techniques. These putative *P. salmonis dot/icm* genes are all transcriptionally active in both tissue culture infected cells and cell-free media. Finally, it has also been demonstrated that the key phagosome-lysosome fusion event is hampered by *P. salmonis,* suggesting that the Dot/Icm system could participate in this process, supporting the relevancy of this secretion system in the potential infection of this novel pathogen.

## Experimental Procedures

### *P. salmonis* Growth Conditions

*P. salmonis* strain LF-89 (ATCC VR-1361) was routinely grown on sheep blood agar plates supplemented with 0.1% L-cysteine and 1% glucose [11], at 23C°. For liquid cultures a single colony of *P. salmonis* was used to inoculate 5 ml of MC1 broth [39], incubating the culture at 23°C with agitation of 100 rpm.

### Degenerate Primer Design

In order to determine the presence of *dot/icm* genes within the *P. salmonis* genome, a set of PCR degenerate primers were designed by comparative analysis using 3 *dot/icm* genes (*dotB*, *dotA* and *icmK*) from phylogenetically related organisms such as *L. pneumophila* and *C. burnetii*. The retrieved sequences were aligned using CLUSTALW (http://www.ebi.ac.uk/clustalw/) [40] and primer properties were validated with the “Oligo Calculator” tool (http://www.basic.northwestern.edu/bio-tools/oligocalc.html). The sequences used in this study were the following: *L. pneumophila icmK* (Gen Bank: AAU26547.1), *C. burnetii icmK* (ABS77467.2), *L. pneumophila dotB* (AAU28734.1), *C. burnetii dotB* (ABX77916.1), *L. pneumophila dotA* (AAA79902.1) and *C. burnetii dotA* (YP_002306135.1). Primer sequences are shown in Table 1.

**Table 1:** Degenerate primers used for the initial amplification of *P. salmonis dotB*, *dotA* and *icmK* genes.

Primer	Sequence	gene
DotB-F1	5'-GCK TCA GAT ATW ACW ATY CAA AC-3'	*dotB*
DotB-R1	5′-TGT TTC AAK AAT RTC GAT RGT-3′	*dotB*
IcmK-F1	5'-ATC GCC GAA AAR MGH RTT CCH CAR-3'	*icmK*
IcmK-R2	5′- GWT GAY ARS ACC ARR TGV CC-3′	*icmK*
DotA-F3	5′-GAY CCR AAR ACY GWW GAA ATY-3′	*dotA*
DotA-R4	5′-GGT CMG GSC GCA TWC KSA G-3′	*dotA*

### Amplification and Sequencing *Dot/icm* Genes

For PCR analysis, *P. salmonis* DNA was extracted from 10 ml MC1 bacterial culture using the AxyPrep™ Multisource Genomic DNA Miniprep Kit (AxyGen Biosciences) in accordance with manufacturer’s instructions. The amplification of all target genes was done with GoTaq Flexi DNA Polymerase (Promega) in a 35-cycle PCR program, using the annealing the corresponding temperature for each primer set. PCR products were visualized on 1% agarose gels stained with GelRed™ (Biotium) and purified with the EZNA Gel Extraction Kit (Omega Biotek) in accordance with manufacturer’s instructions. Purified fragments were cloned into TOPO TA Cloning Kit (Invitrogen) and submitted for sequencing at Macrogen Inc, Korea. DNA sequences were analyzed with BLASTN and BLASTX software (http://blast.ncbi.nlm.nih.gov), limiting the query to bacterial sequences to determine their possible identities. Finally, *P. salmonis* sequences were aligned with analogous sequences obtained from BLAST analysis using CLUSTALW [40] and the alignments were processed using JALVIEW software [41]. Due to the *L. pneumophila* DotA has been reported as a transmembrane protein, an additional analysis using the SOSUI software (http://bp.nuap.nagoya-u.ac.jp/sosui/) was made for the putative *P. salmonis* DotA protein in order to determine transmembrane and hydrophobic domains.

Once the identities of the new *P. salmonis* sequences were obtained, specific primers against the *P. salmonis dotB* gene were designed for Long Range PCR (LR-PCR) purposes, in order to obtain a broader *dot/icm* gene array sequence. LR-PCR was performed using the combinations of the new specific *dotB* primers and the ITS primers (RTS1 and RTS4) [42],as described below: DotB-Forward/RTS1 (ITS Forward), DotB-Forward/RTS4 (ITS Reverse) and DotB-Reverse/RTS1 (ITS Forward). The PCR reactions were carried out with the Pfu Ultra™ high-fidelity DNA Polymerase (Stratagene) under the following conditions: 92°C for 5 minutes followed by 30 cycles of: 92° for 10 seconds, 51°C for 30 seconds and 68°C for 12 minutes. Amplification products were visualized in 1% agarose gel stained with GelRed™. The highest PCR product was selected for sequencing by creating a genome library. The amplicon was digested with the enzyme Sau3AI (New England Biolabs) for 15 minutes at 37°C and the digestion was then ligated to pBluescript SK+ vector (Fermentas) previously linearized with the enzyme BamHI (Promega) and treated with Alkaline Phosphatase (Promega). The ligation reaction was incubated at 22°C for 4 hours in the presence of T4 DNA Ligase (Promega) and the resultant product used to transform chemically-competent *E. coli* TOP10 cells (Invitrogen). Cells were plated on Luria-Bertani (LB) agar supplemented with Ampicillin 100 µg/ml and X-Gal (Promega) and incubated at 37°C overnight. Positive clones were submitted for sequencing at Macrogen Inc., Korea. The sequences were analyzed as described above.

### *P. salmonis* Infection Kinetics in Two Cell Lines

In order to determine the transcriptional activity of the putative *P. salmonis dot/icm* genes during the host infection process, an infection kinetic assay was performed using qRT-PCR for two different cell lines of distinct origin.

The RTS11 cell line (Kindly donated by Dr. Niels Bols, University of Waterloo, Canada) was used as immune cell model since it presents two different cell morphologies: small, round, and non-adherent cells are monocyte-like and large adherent cells with typical macrophage morphology [43]. RTS11 cells were cultured at 20°C in cells in two 25 cm^2^ flasks with Leibovitz’s L-15 medium (Gibco) supplemented with 15% FBS (Gibco).

For a highly proliferative intracellular environment, it has been used the Sf21 insect cell line (Invitrogen) since it has been described for producing high titers of *P. salmonis* at 15 days post-infection [44]. Sf21 cells were maintained at 20°C in 25 cm^2^ flasks in Grace’s Insect Culture Medium (Biological Industries) supplemented with 20% FBS (Gibco).

Both cell lines kinetic infection were measured at earlier stages of the infection (24, 48 and 72 hours). A single colony of *P. salmonis* grown in BCG plates was used to inoculate 3 ml of MC1 medium, incubated at 23°C and 100 rpm until reaching an OD_600_ of 0.6 (12–16 hours approximately). Then, 200 µl of *P. salmonis* medium was used to infect each cell line using one cell flask for every kinetic time point including also a biological duplicate. For the RNA extraction the cells were scraped from the flask, centrifuged at 300 g for 10 minutes. Finally the cellular pellet was processed with Trizol® Reagent (Invitrogen) in accordance to the manufacturer’s instructions. The RNA concentration was measured with a Nanodrop-1000 spectrophotometer and kept al −80°C until use.

### *P. salmonis* Growth Kinetics in Liquid Medium at Acidic pH Levels

The kinetic growth curves were measured for establishing the effect of the acidic pH levels over the transcriptional profile from the *P. salmonis dot*/*icm* genes. For this purpose, 3 ml of MC1 medium was inoculated with a single colony of *P. salmonis* grown in BCG plates, incubating the culture for 16 hours at 23°C and 100 rpm of agitation. Subsequently, this culture was used to inoculate 60 ml of MC1 broth and incubated for 24 hours under the same conditions described above. Six replicas of 50 ml each were started using 5 ml of the previous *P. salmonis* culture as inoculum, incubated at 23°C and 100 rpm. After 12 hours post-incubation the cultures were centrifuged at 6000 rpm for 20 minutes and resuspended in 50 ml MC1 at pH 4.0, 5.5 and 7.0, considering two 2 replica for each condition, and then incubated at 23°C and 100 rpm as usual. Samples from each culture were taken at 2, 4, 6 and 12 hours, centrifuging them at 6000 rpm for 20 minutes and processing the resultant pellets with the Trizol® Reagent (Invitrogen). The RNA was quantified in a Nanodrop-1000 spectrophotometer and kept at −80°C until use.

### QRT-PCR of *P. salmonis* Dot/icm Genes

In order to quantify and compare the expression levels of the putative *dot*/*icm* genes under both cell lines used and growth in MC1 at different pH levels, relative quantification by qRT-PCR was made using as reference gene (housekeeping gene) the ITS (16 S–23 S internal transcribed spacer) for the normalization.

To proceed with the quantification, 2 µg RNA of each sample was pre-treated with 2 units of DNAse RQ1 (Promega) and incubatedat 37°C for 30 min in order to eliminate all putative DNA contamination. Subsequently, the DNAse treated samples were used for cDNA synthesis with M-MLV Reverse transcriptase (Promega) according to the manufacturer’s instructions in the presence of random primers (Promega). The qRT-PCR was performed using 20 µl reaction mixtures containing: 1X of Brilliant III Ultra-Fast SYBR® Green QPCR Master Mix (Stratagene), 300 nM of each primer (see table 2) and 1 µl of template (standard curve or *P. salmonis* cDNA). As a host cell control, the elongation factor 1-alpha (*EF1A*) gene was used to normalize the qPCR and the RNA amount of each kinetic point. Samples were amplified and detected in a CFX96 Real Time PCR System (Biorad) using the following cycle profile for the *dot/icm* genes: 95°C for 3 minutes for initial denaturation; 95°C for 15 seconds followed by 40 cycles of: 58°C for 15 seconds and 60°C for 20 seconds. The primers RTS1 (5′-TGA TTT TAT TGT TTA GTG AGA ATG A-3′) and RTS4 (5′-ATG CAC TTA TTC ACT TGA TCA TA-3′) were used for ITS amplification with a cycling profile of: 95°C for 3 minutes for initial denaturation and 40 cycles of: 95°C for 15 seconds, 51°C for 15 seconds and 60°C for 20 seconds. Primers EF1A-For (5′-GTC TAC AAA ATC GGC GGT AT-3′) and EF1A-Rev (5′-CTT GAC GGA CAC GTT CTT GA-3′) were used for the*EF1A* amplification as described previously [21], using a cycling profile of: 95°C for 3 minutes for initial denaturation and 40 cycles of: 95°C for 15 seconds, 56°C for 15 seconds and 60°C for 20 seconds. In all cases, following the final cycle, melting curve analysis was performed to determine the specificity in each reaction tube (absence of primer dimers and other non-specific products) by heating the samples from 65 to 95°C in 0.5°C increments with a dwell time at each temperature of 5 seconds while continuously monitoring fluorescence. All Real-time PCR were assayed on every biological replicate and each sample was run in duplicate. Additionally, as a negative control qPCR reaction tube of DNAse treated RNA was run for all the experiments in order to confirm that samples were free of DNA contamination.

**Table 2:** Specific primers for *P. salmonis dot/icm* genes used for qRT-PCR.

Primer	Sequence	gene
Ps-DotB-For	5′-GCT ACA TCT CCA TTT CTT GAC CAT TTC-3	*dotB*
Ps-DotB-Rev3	5'- GCA TTA GTG CCG AGC ATT ACA GG-3'	*dotB*
PS-IcmK-F	5′-GCG CCA GAG CAG ATA CAT CAG TAT AAA G-3′	*icmK*
PS-IcmK-R1	5′-GCC ACC GGA ACA TCT AAG CCT TTT AA-3′	*icmK*
Ps-IcmE-For1	5′-GCC TTG GTT AAG TGT GAC CGT TG-3′	*icmE*
PS-IcmE-Rev1	5′-GCT GTC ATT ACC TGC ATT AGA TCA TAG-3′	*icmE*
Ps-DotA-For	5′-GCT TAT GTC GCC ATT TCT GCA GCA CTT C-3′	*dotA*
Ps-DotA-Rev2	5′-CCA CTC ACT CGG CAA ATT AAG CAG-3′	*dotA*

For all cases the real-time PCR efficiencies were calculated from the slope according to the established equation E = 10 ^(−1/slope)^ [45]. The threshold cycle (Ct) values of the CFX Manager Software (Biorad) were transformed to relative quantities as described by Peña et al [21].

For the conversion of the Ct values to relative quantities, reaction efficiencies were used. Relative gene expression for *dotB*, *icmE*, *icmK* and *dotA* were calculated using the values obtained to ITS of each assay as normalization factors. For the expression during infection kinetics the 24 hours post-infection was used as calibrator in both cell lines and for expression in MC1 at different pH the 2 hours of incubation at pH 7.0 was used as calibrator for all genes. Finally, a Mann-Whitney test was used to determine significant differences in gene expression between the biological and experimental replicates and significance was set at P<0.05.

In order to corroborate our results an additional analysis was made for relative quantification using the 2^−ΔΔCt^ method [46]. The ITS data were used for normalization in all cases and the calibrator were the same used in the previous analyses.

### Determination of Phagosome-lysosome Fusion

To determine that *P. salmonis* is able to fuse with lysosomes after bacterial phagocytosis, we performed immunofluorescence staining with three different infected-cell lines (RTS11, Sf21 and CHSE-214). RTS11 and Sf21 cells were cultured as described above and CHSE-214 cells (ATCC CRL 1681) were maintained in MEM medium (Gibco) supplemented with 15 mM HEPES, 10 mM sodium bicarbonate, and 10% FBS (Gibco) at 17°C [9].

For immunofluorescence, the cells were cultured in 6 well plates with glass coverslips previously treated with 0.1% of L-Polylysine (Sigma-Aldrich) diluted in sterile ultrapure water. After 7 days of incubation, the cells were infected with 50 µl of 12 hour-old *P. salmonis* culture and incubated for 5 days. As a control, 1 ml of12 hour *P. salmonis* culture was inactivated with 3% formaldehyde (Sigma-Aldrich) for 24 hours at 4°C, washed 5 times in PBS 1X after inactivation and finally resuspended in 1 ml of sterile PBS 1X. Finally, 50 µl of inactivated *P. salmonis* culture was used to infect one well of a CHSE-214 titer plate, and incubated for 48 hours. The infected cells with *P. salmonis* live and/or dead were subsequently incubated at 20°C for 3 hours in darkness with 75 nM of LysoTracker Red DND-99 (Invitrogen) diluted in the medium used for each cell line and then washed three times with PBS 1X sterile. The cells were fixed for 30 minutes in 3% paraformaldehyde in PBS 1X at pH 7.5 and immediately permeabilized with 0.1% Triton X-100 (Sigma-Aldrich) for 20 minutes and washed 3 times with PBS 1X. The infected cells were incubated in the dark for 30 minutes at 20°C with a 1∶100 (v/v) dilution in BSA 3% in PBS 1X of the FITC-conjugated oligoclonal antibody anti-*P. salmonis* (SRS Flourotest, BiosChile). Afterwards, cells were washed three times with PBS 1X, mounted with Dako® mounting medium (Invitrogen). The samples were analyzed using a Leica TCS SP5 II Spectral Confocal Microscope (Leica Microsystems Inc.). The images were obtained with a Leica 40x/1.25 Oil HCX PL APO CS objective (Leica Microsystems Inc.).

## Results and Discussion

### *P. salmonis* Encoded ORFs *dot/icm* Homologue

Based upon on the available sequences TIVSS Dot/Icm genes of the pathogens *L. pneumophila* and *C. burnetii*, degenerate primers were designed over conserved regions of 10 out of 22 possible ORF counterparts. A preliminary screening yielded PCR-positive amplicons corresponding to three key genes: *dotB, dotA* and *icmK*, which were subsequently cloned into pCR 2.1 TOPO TA vector and submitted to sequence. A fourth putative gene, *icmE,* was obtained using LR-PCR. Interpretation and significance of the DNA sequences was analyzed using three different bioinformatics tools: BLASTN, which determines nucleotide sequence homologies; BLASTX, which interprets the protein-coding potential of the target sequence and ClustalW, which aligns and provides amino acid homologies of the presumed proteins.

**Figure 1:**
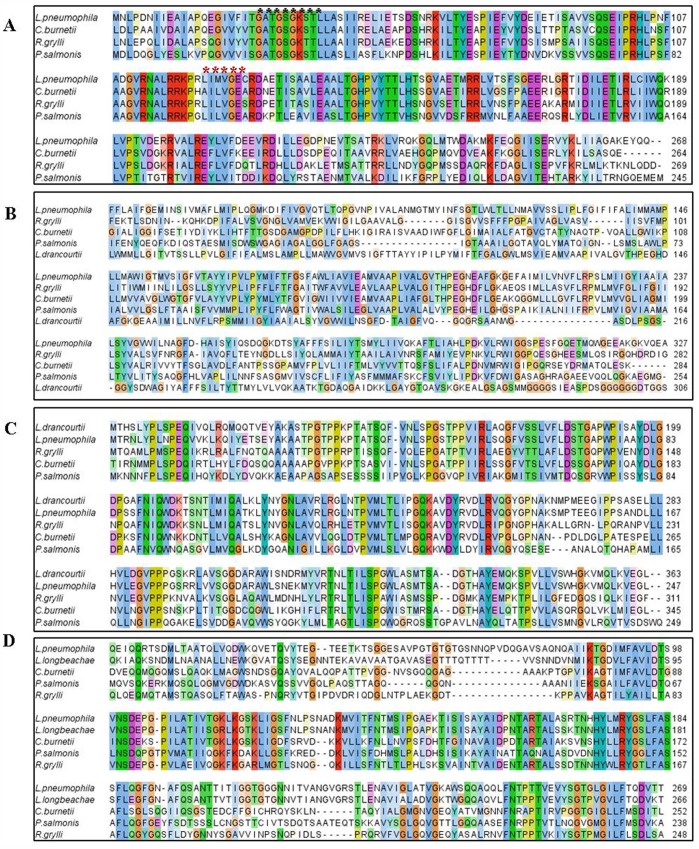
ClustalW alignments among the most conserved regions of *P. salmonis dot/icm* protein products with their homologues. A: DotB alignment, where the black and red asterisks show the Walker A and B, respectively (ATP binding site); **B:** DotA alignment; **C:** IcmK alignment; **D:** IcmE alignment. All figures were created using Jalview, where the color intensity shows the conservation degree of the aminoacids between the sequences.

Regarding the putative *P. salmonis dotB* gene (Gene Bank: JX477678), BLASTN *s*howed 69% and 68%of identity with *L. pneumophila* and *C. burnetii*, respectively. BLASTX analyses confirmed an in-frame ORF encoding a protein homologous to the DotB ATPase of the Dot/Icm secretion system of the two same reference pathogens mentioned above and also resemble to ATPase sequences from an array of different microorganisms (Table 3). About ClustalW alignment, this putative protein showed high degree of conservation with its counterparts (Figure 1–A). Furthermore, *P. salmonis* DotB contains two conserved distinctive motifs described for the P-loop NTPase superfamily: the first, consisting of a nucleotide phosphate-binding site, also known as the Walker A motif (GxxxxGK[S/T]); and the second, the Walker B motif (hhhh[D/E), where “h” is a hydrophobic residue [47]. The Walker A and B motifs bind the beta-gamma phosphate moiety of the donor molecule (either ATP or GTP) and the Mg2+ cation, respectively [48]. For the *P. salmonis* analogue, the putative Walker A motif involves the sequence GATGSGKS which fully matches the sequence of their reference counterparts, while the Walker B sequence (LILVGE) although not identical, does share the same physiochemical features as its counterparts (Figure 1–A). Nowadays, the *L. pneumophila* DotB is the best characterized protein, which fulfills three associated complementary functions: i) participation in the assembly of the TIVSS; ii) protein exportation; and iii) pilus retraction [49]. In addition, the prototype protein shares several structural features with type II and type IV secretion ATPases, which should also be present in the putative *P. salmonis* protein. One of such feature is an important hexameric ring structure that is believed to concede a loose association with the bacterial inner membrane despite its hydrophilic nature [49], [50], [51], [52].

**Table 3:** BLASTX results for *P. salmonis dotB, dotA, icmK* and *icmE* genes.

GenBank N°	Organism	Protein	ID %	“e” Value
EDP46591.1	*Rickettsiella grylli*	DotB	69	2.0E–40
CBJ10695.1	*Legionella longbeachae* NSW150	DotB	69	5.0E–39
ABX77916.1	*Coxiella burnetii* RSA	DotB	68	5.0E–38
AAU28734.1	*Legionella pneumophila*	DotB	68	3.0E–38
EHL29908.1	*Legionella drancourtii* LLAP12	DotA	33	1.0E–24
ABX79068.1	*Coxiella burnetii* RSA	DotA	31	1.0E–24
AAP75479.1	*Legionella pneumophila*	DotA	29	2.0E–22
EDP46828.1	*Rickettsiella grylli*	DotA	27	3.0E–20
CAA75165.1	*Legionella pneumophila*	IcmE	45	5.0E–33
CBJ13218.1	*Legionella longbeachae* D-4968	IcmE	45	6.0E–32
EDP45922.1	*Rickettsiella grylli*	IcmE	41	2.0E–30
EDR35505.1	*Coxiella burnetii* RSA	IcmE	36	1.0E–30
EHL29524.1	*Legionella drancourtii* LLAP12	IcmK	49	1.0E–30
AAS91991.1	*Legionella pneumophila*	IcmK	49	3.0E–30
EDR35502.1	*Coxiella burnetii* RSA	IcmK	46	5.0E–29
EDP46565.1	*Rickettsiella grylli*	IcmK	41	3.0E–28

The table shows proteins obtained with query coverage above 90 and e value of e−25.

Concerning to the *dotA* and *icmK* analogues, positive *P. salmonis* amplicons did not show sequence similarities via BLASTN analyses. Nevertheless, BLASTX analyses did reveal two putative proteins highly homologous to those expected for the DotA and the IcmK ORFs (Table 3). Additionally, Figures 1B and 1C show the ClustalW protein alignment of the putative *P. salmonis* DotA (JX477679) and IcmK (JX477681) sharing reasonable sequence similarity or identity percentage with their reference counterparts. DotA from *L. pneumophila* is an integral 113 kDa cytoplasmic membrane protein although it is exported by the Dot/Icm system during bacterial growth in liquid media [53]. It contains eight hydrophobic domains and is essential in regulating initial phagosome trafficking towards fusion to the lysosome, a pivotal early decision taken by the pathogen after macrophage uptake [54]. Similar to the *L. pneumophila* protein, the SOSUI analysis of the putative *P. salmonis* DotA reveals the presence of at least five hydrophobic domains with 23 residues each one, related with transmembrane regions (Table 4), indicating that this proteins could be associated with the bacterial membrane. The relevancy of *L. pneumophila* DotA is demonstrated by the fact that defective mutants are unable to inhibit the phagosome-lysosome fusion, which impedes productive infection [55].

**Table 4:** Hydrophobic domains of the *P. salmonis* DotA protein obtained using the SOSUI software.

N terminal	Transmembrane region	C terminal	Type	Length
275	ALGGLFGAGSIGTAAAILGQTAV	297	SECONDARY	23
313	AWLPIALVVLGSLFTAAISFVVM	335	PRIMARY	23
349	IVWALSILEGLVAAPLVALALVY	371	PRIMARY	23
389	LNIIFRPVLMVIGVIAAMALTYV	411	PRIMARY	23
435	GMVNGIVSCFLIFIYASFMMMAF	457	PRIMARY	23

Can be detected 5 putative transmembrane domains.

On the other hand, the *L. pneumophila* IcmK (DotH) protein is a periplasmatic and outer-membrane protein that possesses a peptide secretion signal and constitutes the scaffold core structure of the Dot/Icm complex in association with DotC, DotD, DotF and DotG [56], [57]. Its function is centered on pore formation and is crucial for macrophage killing [58].

The fourth putative gene, the *icmE* (JX477680) was obtained after sub cloning a 12 Kb LR-PCR amplicon. BLASTX analyses of a number of clones obtained from the LR-PCR yielded only one with the expected ORF, displaying high identity values with the IcmE protein of *L. pneumophila, C. burnetii* and *R. gryllii* (Table 3). ClustalW alignment of the *P. salmonis* IcmE protein confirmed the expected homology with other bacterial counterparts (Figure 1–D). In addition, the putative *P. salmonis* IcmE protein has conserved domains with the conjugational protein TrbI, similar to its counterpart in *L. pneumophila,* which also shares sequence homology with a plasmid-encoded protein TrbI from the IncP plasmid RK2 [59]. The main characteristic of the *L. pneumophila* IcmE or DotG (inner membrane protein) is that it interacts with other components of the complex TIVSS, triggering the assembly of the ATP-dependent export channel. The cascade involves DotF, which associates with outer membrane proteins IcmK, DotC and DotD to transfer energy from ATP hydrolysis to the outer membrane [57]. In general, the characterization of the IcmE analogue clearly suggests the existence of a functional Dot/Icm-like system in *P. salmonis*, which is sustained by the importance these genes have in other pathogenic bacteria.

### *Dot/Icm* Gene Expression during *in vitro* Infection

**Figure 2:**
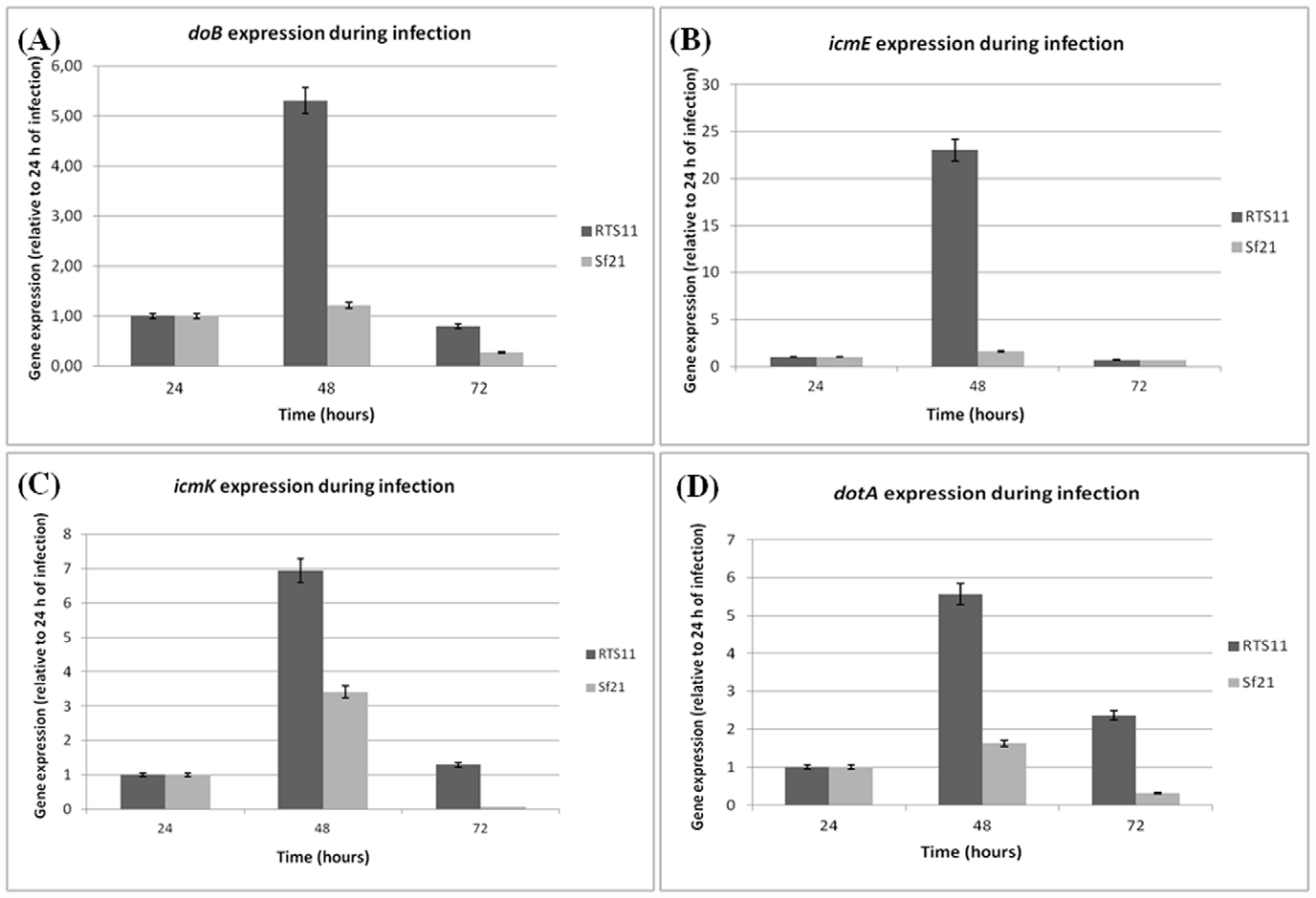
Expression profile of *P. salmonis dot/icm* genes during RTS11 and Sf21cell lines kinetic infection. Gene expression was determined by qRT-PCR, using relative quantification. **A:** *dotB* gene expression number; **B:** *icmE* gene expression; **C:** *icmK* mRNA gene expression; **D:** *dotA* gene expression. Gene expression was normalized by the use of ITS like a housekeeping gene. 24 hours post-infection in each cell line was used as calibrator (value = 1).

The evidences shown above suggest that *P. salmonis* comprises a putative Dot/Icm system including structural and proton-driving elements. If the putative *P. salmonis dot/icm* genes (TIVSS) were involved in modulating biogenesis of the vacuole, where the bacterium replicates inside the cells, these genes should be expressed during the infection process. Thus, the kinetic infection was evaluated at early stages of infection (24, 48 and 72 hours) in both cell lines: the phagocytic fish-derived RTS11 cell line and the non-phagocytic insect-derived Sf21 cell line. qRT-PCR analysis of *dotB*, *icmK*, *icmE* and *dotA* genes showed high levels of expression for at least the first three days after challenging (Figure 2). Our result shows a behavior is similar to that displayed by *Brucella suis,* where TIVSS genes are induced within the first few hours after bacterial uptake by macrophages [26]. In fact, the *P. salmonis dot/icm* gene expression was notoriously higher in the macrophage derived cell line (RTS11) with an evident overexpression at 48 hours with values of 5.31 for *dotB,* 23.04 for *icmE,* 6.96 for *icmK* and 5.57 for *dotA* (Figure 2). Nevertheless, expression of all *dot/icm* genes was higher at 48 hours post-infection in both cell lines, but the values obtained for the Sf21 cells were noticeably lower than RTS11 data (Figure 2), indicating that overexpression of these genes is preferentially triggered in macrophages. The high expression of *dot/icm* genes at 48 hours post-infection could be explained because *P. salmonis* grows at a slow rate in tissue culture cells and the major bacterial uptake by host cells may peak at 48 hours after infection and therefore expression of the *dot/icm* genes would peak at this time. A similar event has been observed in *C. burnetti*, which also displays low growth rates *in vitro*, and the expression of *dotB* and other Dot/Icm markers is not detected before 24 hours of infection, only at 48 hours post-infection the bacterium begin to appear in large vacuoles inside the cells [60]. Additionally, normalization with the 2^−ΔΔCt^ method was made, obtaining similar results for all genes in both cell lines and the same tendency of expression was detected (Figure S1). While the *dot/icm* gene expression peaks at 48 hours post-infection, the ITS expression peaks at 72 hours in both cell lines and it was possible to detect an increasing ITS transcript in time (data not shown), possibly due to the multiplication of *P. salmonis* in both cell lines, which led to an increase in the number of transcripts. This result has validated our previous data, suggesting a differential expression in time of the secretion genes in earlier stages of infection. Negative RNA controls confirm a non-DNA contamination in the samples used for cDNA synthesis (data not shown). Furthermore, the *EF1A* gene expression on both cell lines demonstrate a constant amount of host cells during the kinetics (at least at earlier stages of infection) with Ct values of 30 and 32 for RTS11 and Sf21, respectively (Table S1) and consequently the eukaryotic mRNA is constant.

**Figure 3:**
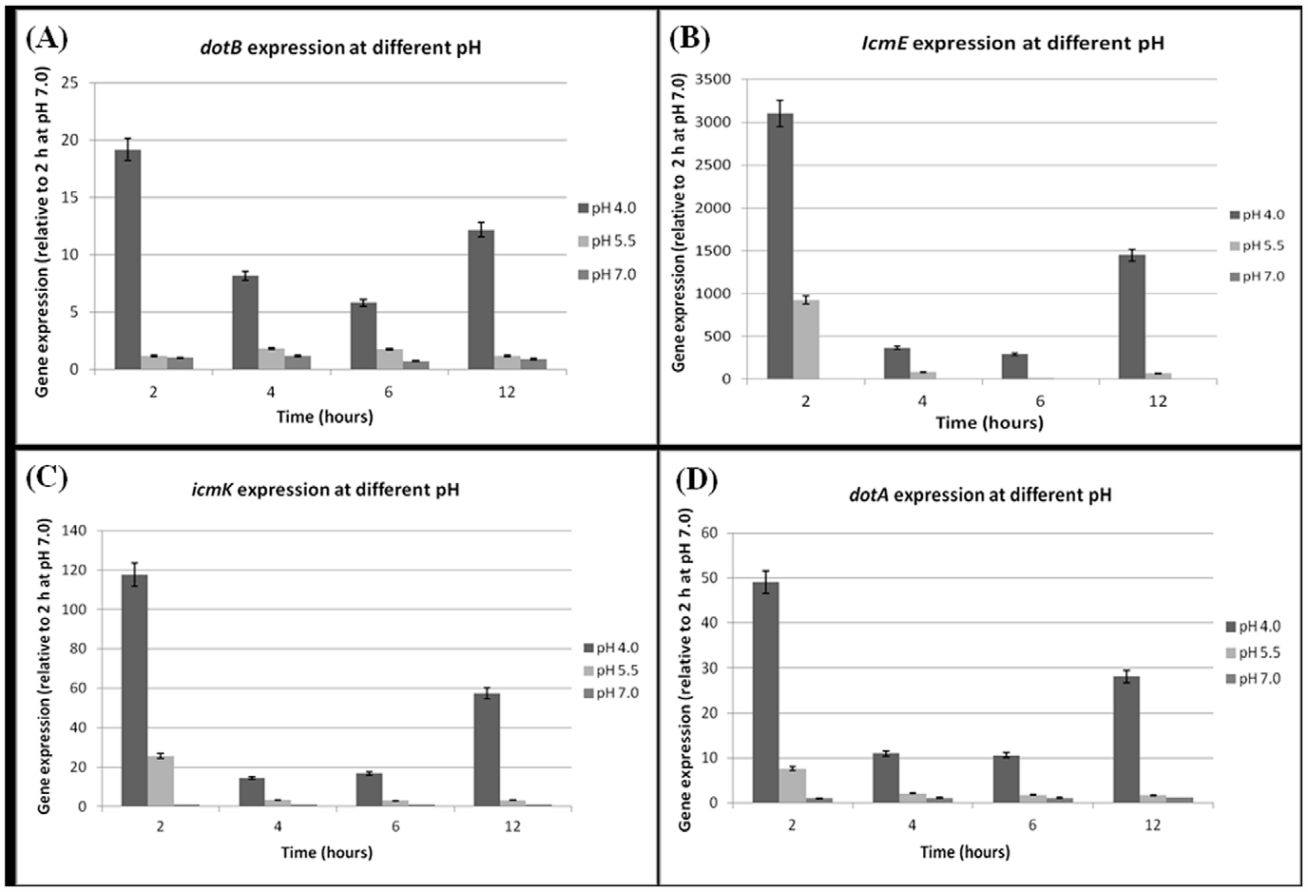
Expression profiles of *dot/icm* genes throughout *P. salmonis* growth kinetics at different pHs in MC1 medium. Gene expression was determined by qRT-PCR, using relative quantification. **A:** *dotB* gene expression number; **B:** *icmE* gene expression; **C:** *icmK* gene expression; **D:** *dotA* gene expression. Gene expression was normalized by the use of ITS like a housekeeping gene. Two hours of growth at pH 7.0 was used as calibrator (value = 1) for all genes. The figure shows that all genes were notably over-expressed at pH 4.0, particularly at 2 hours of incubation.

*P. salmonis* expresses *dot/icm* genes either in cell culture or in cell-free media (Figures 2 and 3). Comparatively, the Vir-like TIVSS genes in *Brucella abortus* and *Brucella melitensis* are constitutively expressed independent of the environment [61]. However, *Brucella suis*is unable to express the corresponding genes when growing in liquid media [62], suggestive that their expression might be responsive to limiting stress conditions.

### Acid pH Induces Overexpression of *dot/icm* Genes in *P. salmonis*

We hypothesized that phagosome acidification could be the signal that induces an intracellular over-expression of the *P. salmonis dot/icm* genes. So as to confirm this hypothesis, expressions of the *dot*/i*cm* genes were evaluated by growth kinetics at pH 4.0, pH 5.5 and neutral pH.

qRT-PCR results show that *dot*/*icm* genes are clearly induced at acidic medium, specifically at pH 4.0 with values peaking at 2 hours of incubation (Figure 3). As seen in the figure, the lower the pH the higher the induction obtained. In comparison with expression at pH 7.0 at 2 hours of incubation, the induction at pH 4.0 increase 25-fold for *dotB*, 3000-fold for *IcmE*, 117-fold to *icmK* and 49-fold to *dotA*. At pH 5.5 the expression levels were higher than pH 7.0 but lower than pH 4.0, with values of 1.4, 900, 25 and 7.6 for *dotB, icmE, icmK* and *dotA* respectively. A similar expression tendency was observed for all genes using the 2^−ΔΔCt^ normalization method (Figure S2), validating our previous data. As a control, ITS transcription (estimated by Ct values) remained stable at all times during the kinetics, except when the bacteria were grown at pH 4.0 with a decrease in 2 Ct units at 12 hours (Table S2), wherein the *dot/icm* expression is higher than at 4 and 6 hours (Figure 3), indicating that general protein expression is diminished at acidic pH, while *dot/icm* expression is promoted. A similar phenomenon is observed in *B. suis*, where 3 hours acid shock at pH 4.0 reduced the levels of protein synthesis while transcription of the TIVSS operon is strongly induced [63], [26]. Our data are consistent with the idea that acid pH induces over-expression of *dot*/*icm* genes, supporting our rationality where phagosome acidification could represents a crucial event in triggering expression of these genes during *P. salmonis* infection and could help as well to the intracellular survival along with other potential virulence mechanisms, at least initially at acid pH inside the cells.

### *P. salmonis* Inhibition of Phagosome-lysosome Fusion

Phagocytosis is a process mediated by binding of organisms or large particles to plasma membrane receptors on phagocytes followed by internalization in newly formed phagosomes, organelles which mature into acidic and protease-rich phagolysosomes, where phagocytosed microorganisms or materials are killed and/or degraded [64]. In macrophages, phagosome-lysosome membrane fusion is a tightly regulated event essential for intracellular microorganism killing [65]. For this reason, many bacterial pathogens have developed strategies, which allow them to evade phagosome-lysosome fusion to survive and multiply within the intracellular environment [66]. For example, *B. abortus* is able to replicate in a compartment segregated from the endocytic pathway and the maturation of the *Brucella*-containing vacuole involves sustained interactions and fusion with the endoplasmic reticulum (ER), which creates a replicative compartment with ER-like properties, in a TIVSS-dependent process [67]. *Bartonella henselae* and *S. typhimurium* have a specific competence to actively avoid the host endocytic pathway after entry into macrophages and epithelial cells, from within a specialized non-endocytic membrane-bound vacuole is formed in a TIVSS- and TIIISSS-dependent event, respectively [66], [68], [69].

**Figure 4:**
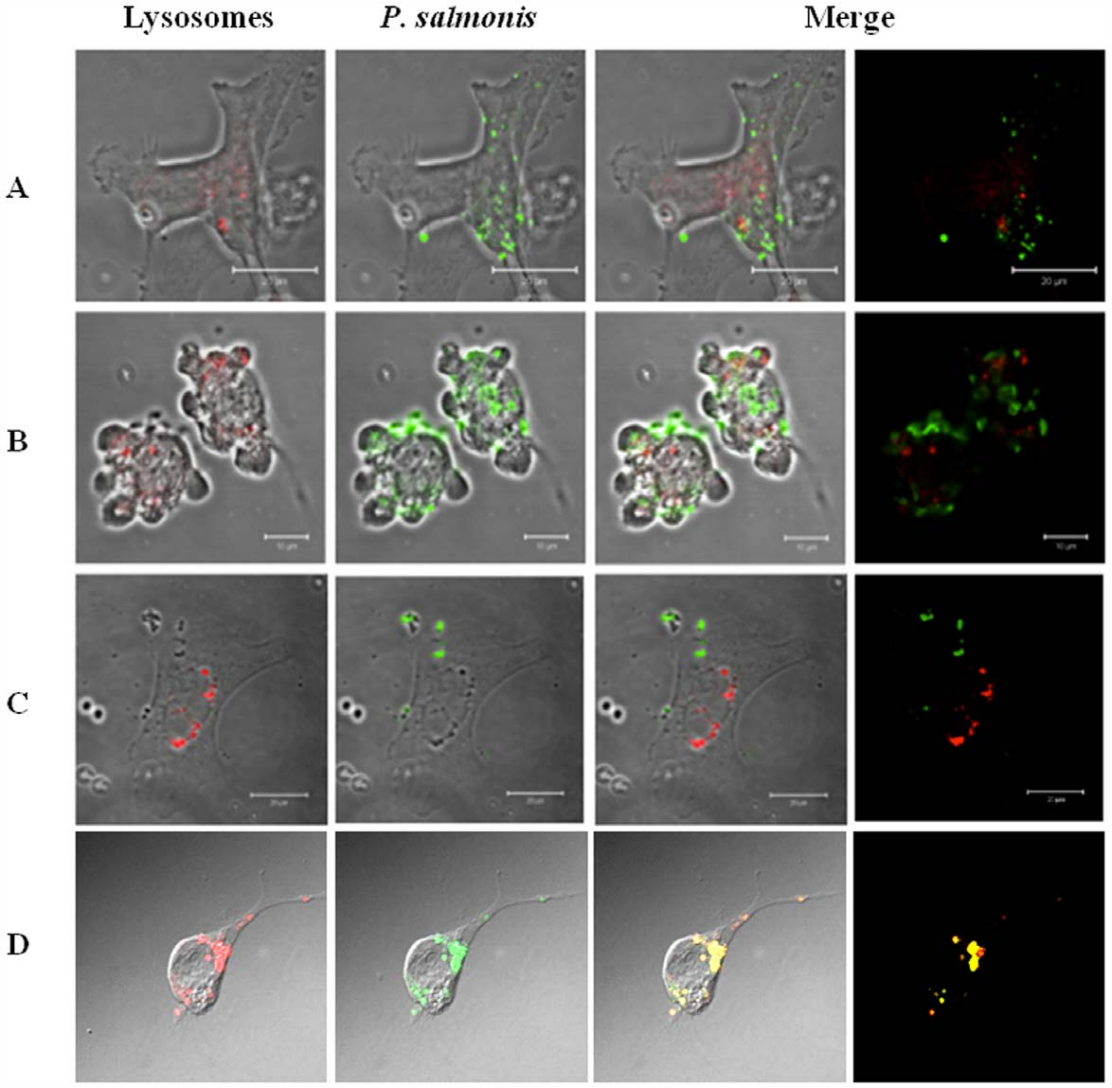
Confocal Laser Scanning Microscopy of *P. salmonis* infection on three cell lines showing the escape of phagosome-lysosome fusion. The immunofluorescence was made 5 days post-infection. Lysosomes were stained in red with LysoTracker Red reagent and *P. salmonis* was detected with a FITC conjugated antibody. **A:** CHSE-214 cell line infected with *P. salmonis*. **B:** Sf21 cell line infected with *P. salmonis*. **C:** RTS11 cell line infected with *P. salmonis*. **D:** CHSE-214 cell line infected with formaldehyde-inactivated *P. salmonis*, the immunofluorescence stain was made at 48 hours after infection.

As our data support the possibility that *P salmonis dot*/*icm* genes could play a pivotal role in the biogenesis of the replicative vacuole, we decided to determine whether a phagosome-lysosome escape mechanism exists in *P. salmonis* infected cells. Three different cell lines, the non-phagocytic insect (Sf21), a second non-phagocytic salmonid embryo kidney (CHSE-214) and the phagocytic trout macrophage/monocyte (RTS11), were infected for 5 days because at this time the typical *P. salmonis-*induced Cytopathic Effect (CPE) is fully evident in all cell lines used, confirming the viability of the bacterium and therefore is an evidence of productive infection. After day five, the infected cells were stained with a lysosomal-specific probe (LysoTracker^(^™^)^ Red) to detect the organelle and also with aspecific FITC-labeled anti-*P salmonis* to evaluate possible co-localization with the phagosome-containing bacteria. Samples were then analyzed under a Laser Scanning Confocal Microscope. As seen in Figure 4, phagosome-lysosome fusion does not occur in any of the three cell lines, indicating that *P. salmonis* could use this strategy to promote its replication inside isolated vacuole. In contrast, the control where formaldehyde inactivated bacterium was used to infect the RTS11 cell line, showed clearly that the inactive *P. salmonis* is unable to evade the phagosome-lysosome fusion and consequently is derived directly to the endocytic pathway for being degraded. This final result is consistent to sustain that *P. salmonis* is able to evade the phagosome-lysosome fusion event that could be targeted by the expression and effectors secretion of the Dot/Icm system and/or by another virulence mechanism to ensure their multiplication in the infected cells.

Comparatively, *L.pneumophila* and *C. Burnetii*are known to depend on a similar pathway in which an initial generation of an organelle-like structure inside the host cell helps them support bacterial replication [70], [60]. Therefore, it is feasible to think that due to the similarities shared by these two pathogens with *P. salmonis*, the latter could use the same strategy to avoid phagosome-lysosome fusion, and if so, *dot/icm* gene products should hold this responsibility.

## Final Remarks

Most intracellular and facultative pathogens employ TIVSS as a preferential mechanism to direct biogenesis of a vacuolar replicative niche that circumvents default maturation through the endolysosomal cascade and favors their intracellular multiplication [71]. This also seems to be the case for *Piscirickettsia salmonis.* Out of the two existent ancestral lineages of the TIVSS: the VirB/D4 system of *A. tumefaciens* and the Dot/Icm system of *L. pneumophila*, sometimes referred as the TIVSS-A and TIVSS-B systems, respectively [72], *P. salmonis* clearly uses the latter. Existing evidence suggests that proteins translocated by the Dot/Icm system are critical for successful parasitism of macrophages by either *C. burnetii* and/or *L. pneumophila* [73]. In this report, we have demonstrated the functional existence of four essential components of Dot/Icm secretion system (*dotB*, *dotA*, *icmK* and *icmE*) in the genome of fish pathogen *P. salmonis*. Moreover, the putative corresponding polypeptide products also share distinctive features with their counterparts, corroborating our interpretation that the system exists, in spite of the fact that not all presumptive components have been characterized. Our results also indicate that these genes are expressed during the infection as well as in growing cell-free medium, suggestive of a constitutive expression in response to the stress signaling compatible with the rough environment the bacteria faces *in vivo*. In addition, qRT-PCR experiments show that the *dot/icm* genes are over expressed in acid pH, indicating possibly that phagosome acidification is the triggering event that produces *dot/icm* gene expression and therefore protein secretion via the Dot/Icm system to favor intracellular bacterial replication. Finally, we have also demonstrated that *P. salmonis*-containing vacuoles do not fuse with lysosomes, indicating that there is a bacterium-driven interference in the endosomal maturation process that ensures bacterial survival, of which the Dot/Icm secretion system should be responsible by delivering effectors proteins inside the host cell.

Hence, we may conclude that the *P. salmonis* Dot/Icm secretion system represents one of the mechanisms associated with its virulence and pathogenesis, similar to what happen with the closely related pathogens *L. pneumophila* and *C. burnetii*. In order to demonstrate this action, *knock out* gene experiments could be carried out once *P. salmonis* be efficiently transformed.

## Supporting Information

Figure S1 *P. salmonis dot/icm* gene expression during an infection kinetic in RTS11 and Sf21 cell lines. The normalization was made using the 2^−ΔΔCt^ method. **A:** *dotB* gene expression number; **B:** *icmE* gene expression; **C:** *icmK* mRNA gene expression; **D:** *dotA* gene expression. Gene expression was normalized by the use of ITS like housekeeping gene. 24 hours post-infection in each cell line was used as calibrator (value = 1).
(DOC)

Figure S2 *P. salmonis dot/icm* gene expression during a growth kinetic at different pH. The normalization was made using the 2^−ΔΔCt^ method. **A:** *dotB* gene expression number; **B:** *icmE* gene expression; **C:** *icmK* mRNA gene expression; **D:** *dotA* gene expression. Gene expression was normalized by the use of ITS like a housekeeping gene. Two hours of growth at pH 7.0 was used as calibrator (value = 1).
(DOC)

Table S1 *EF1A* gene Ct values of RTS11 and Sf21 cell lines during infection kinetics determined by qRT-PCR.
(DOC)

Table S2 ITS (16 S-23 S internal transcribed spacer) Ct values obtained during *P. salmonis* growth kinetic at different pH.
(DOC)
