# Supplementary figures and images for: Evidence of the Presence of a Functional Dot/Icm Type IV-B Secretion System in the Fish Bacterial Pathogen Piscirickettsia salmonis
Source: PLoS One. 2013 Jan 28;8(1):e54934. doi: 10.1371/journal.pone.0054934 (PMC3557282; doi:10.1371/journal.pone.0054934)

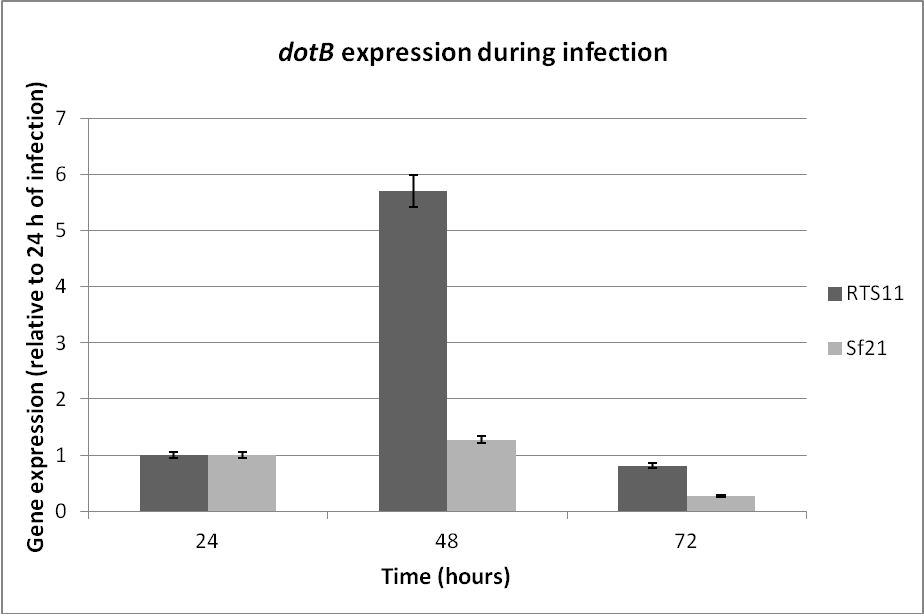

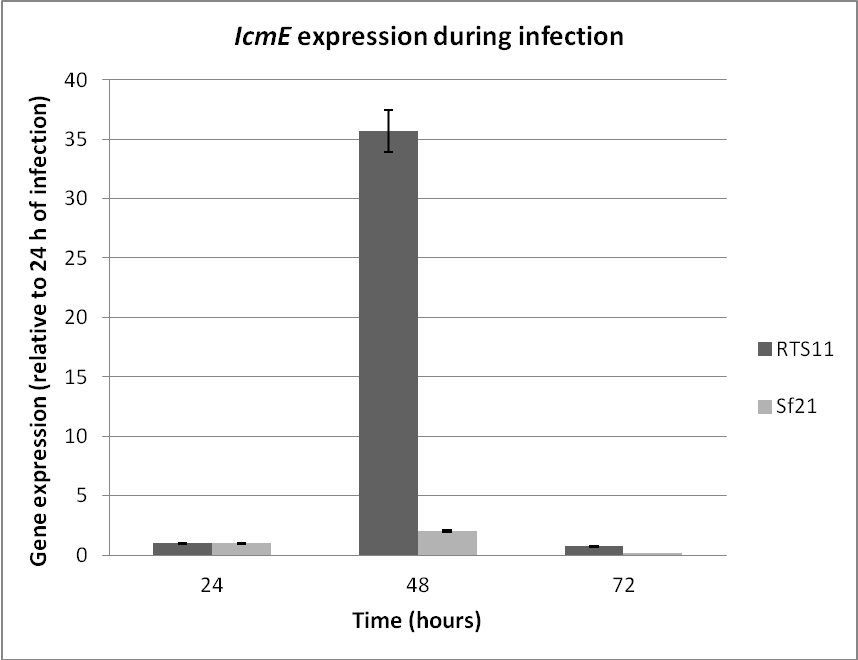

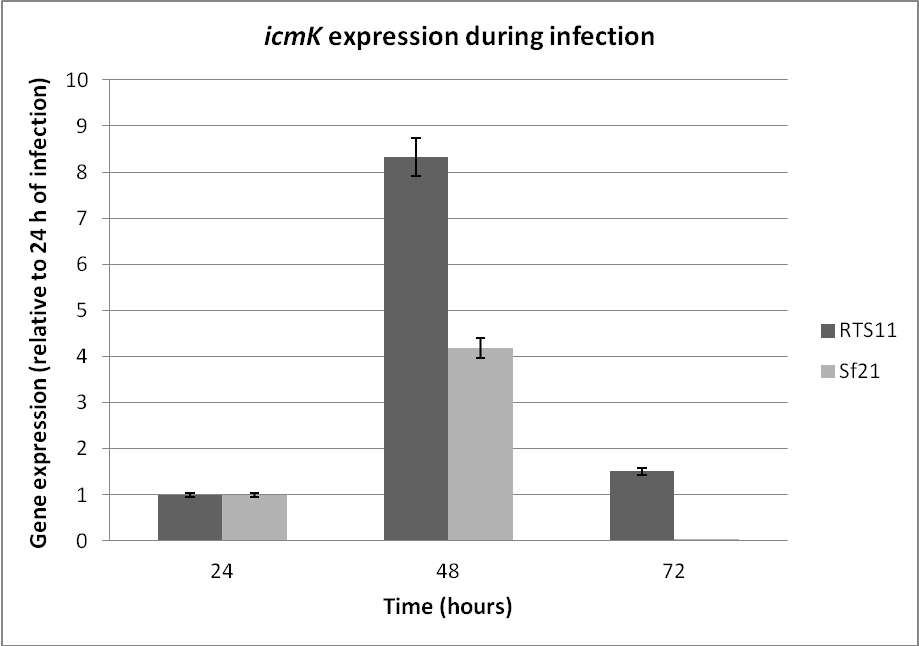

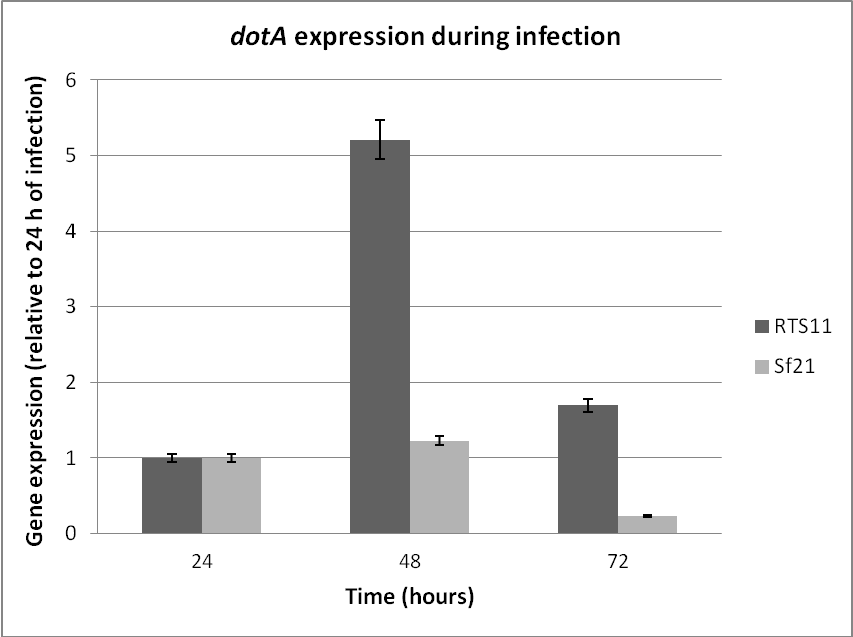


**A**

**B**

**C**

**D**

Supplement: Figure S1 — P. salmonis dot/icm gene expression during an infection kinetic in RTS11 and Sf21 cell lines. The normalization was made using the 2−ΔΔCt method. A: dotB gene expression number; B: icmE gene expression; C: icmK mRNA gene expression; D: dotA gene expression. Gene expression was normalized by the use of ITS like housekeeping gene. 24 hours post-infection in each cell line was used as calibrator (value = 1). (DOC) [file pone.0054934.s001.doc]

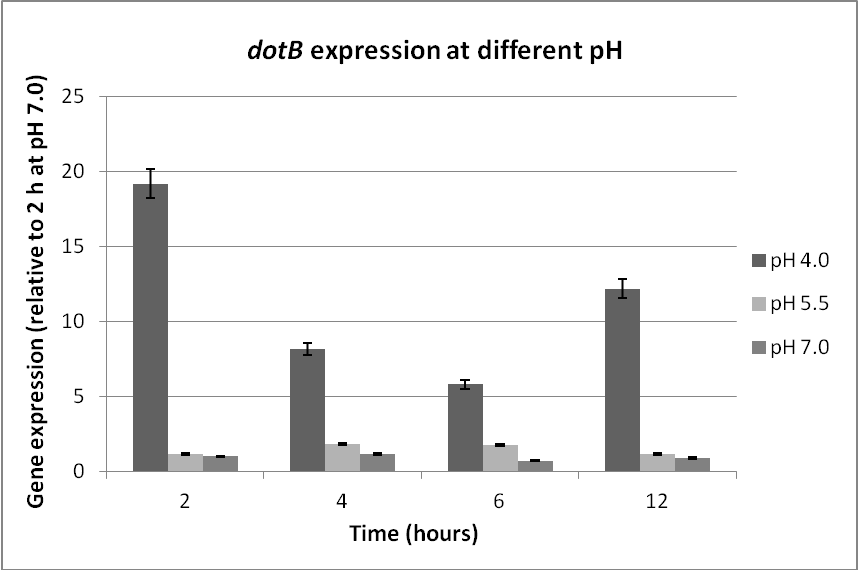

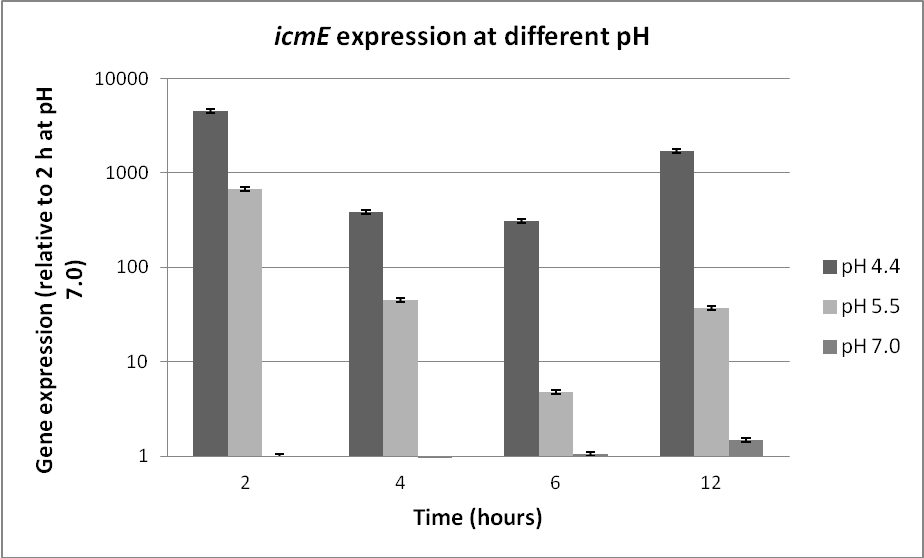

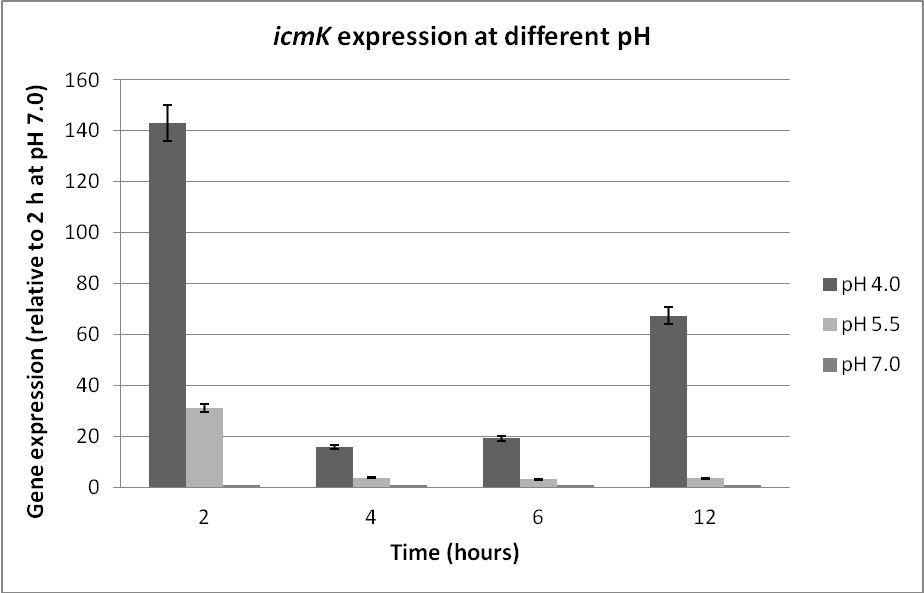

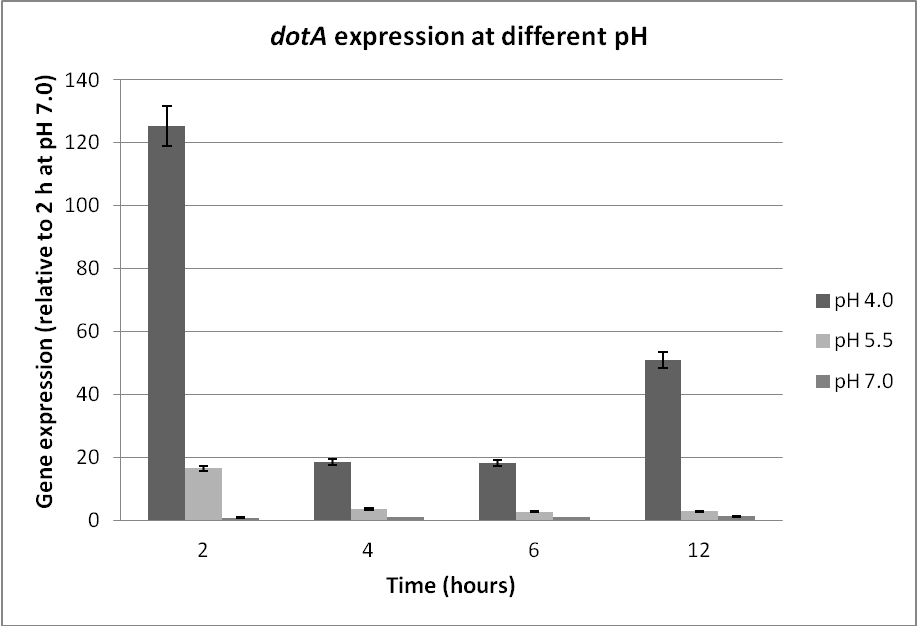


**A**

**B**

**C**

**D**

Supplement: Figure S2 — P. salmonis dot/icm gene expression during a growth kinetic at different pH. The normalization was made using the 2−ΔΔCt method. A: dotB gene expression number; B: icmE gene expression; C: icmK mRNA gene expression; D: dotA gene expression. Gene expression was normalized by the use of ITS like a housekeeping gene. Two hours of growth at pH 7.0 was used as calibrator (value = 1). (DOC) [file pone.0054934.s002.doc]
