# Supplementary material for: Evidence of the Presence of a Functional Dot/Icm Type IV-B Secretion System in the Fish Bacterial Pathogen Piscirickettsia salmonis
Source: PLoS One. 2013 Jan 28;8(1):e54934. doi: 10.1371/journal.pone.0054934 (PMC3557282; doi:10.1371/journal.pone.0054934)
Supplement: Table S1 — EF1A gene Ct values of RTS11 and Sf21 cell lines during infection kinetics determined by qRT-PCR. (DOC) [file pone.0054934.s003.doc]

| **Cell line /Infection time** | **Ct of Biological Replicate 1** | **Ct of Biological Replicate 2** | **Ct of Biological Replicate 3** | **Standard Deviation** |
| --- | --- | --- | --- | --- |
|
| RTS11 24 hours | 30,13 | 30,02 | 30,11 | 0,059 |
| RTS11 48 hours | 30,23 | 30,04 | 29,98 | 0,131 |
| RTS11 72 hours | 29,96 | 30,71 | 30,14 | 0,392 |
| Sf21 24 hours | 32,04 | 32,09 | 32,29 | 0,134 |
| Sf21 24 hours | 31,26 | 31,22 | 31,84 | 0,349 |
| Sf21 72 hours | 31,41 | 32,29 | 33,77 | 1,193 |
