# Supplementary material for: Evidence of the Presence of a Functional Dot/Icm Type IV-B Secretion System in the Fish Bacterial Pathogen Piscirickettsia salmonis
Source: PLoS One. 2013 Jan 28;8(1):e54934. doi: 10.1371/journal.pone.0054934 (PMC3557282; doi:10.1371/journal.pone.0054934)
Supplement: Table S2 — ITS (16 S-23 S internal transcribed spacer) Ct values obtained during P. salmonis growth kinetic at different pH. (DOC) [file pone.0054934.s004.doc]

| **pH** | **Ct 2 hours** | **Ct 4 hours** | **Ct 6 hours** | **Ct 12 hours** |
| --- | --- | --- | --- | --- |
| **4.0** | 17,68 | 18,34 | 18,42 | 19,57 |
| **5.5** | 16,17 | 15,97 | 16,19 | 15,63 |
| **7.0** | 18,45 | 18,62 | 18,53 | 18,49 |
